# Cruciferous vegetable consumption and the risk of pancreatic cancer: a meta-analysis

**DOI:** 10.1186/s12957-015-0454-4

**Published:** 2015-02-12

**Authors:** Li-yi Li, Yue Luo, Ming-dong Lu, Xiao-wu Xu, Hai-duo Lin, Zhi-qiang Zheng

**Affiliations:** Department of General Surgery, The Second Affiliated Hospital & Yuying Children’s Hospital of Wenzhou Medical University, Wenzhou, Zhejiang Province 325027 China; Department of Pediatrics, The Second Affiliated Hospital & Yuying Children’s Hospital of Wenzhou Medical University, Wenzhou, China

**Keywords:** Cruciferous vegetables, Diet, Epidemiology, Meta-analysis, Pancreatic cancer

## Abstract

**Background:**

Previous studies regarding the association between cruciferous vegetable intake and pancreatic cancer risk have reported inconsistent results. We conducted a meta-analysis to demonstrate the potential association between them.

**Methods:**

A systematic literature search of papers was conducted in March 2014 using PubMed, EMBASE, and Web of Science, and the references of the retrieved articles were screened. The summary odds ratios (ORs) with 95% confidence interval (CI) for the highest versus the lowest intake of cruciferous vegetables were calculated.

**Results:**

Four cohort and five case–control studies were eligible for inclusion. We found a significantly decreased risk of pancreatic cancer associated with the high intake of cruciferous vegetables (OR 0.78, 95% CI 0.64–0.91). Moderate heterogeneity was detected across studies (*P* = 0.065). There was no evidence of significant publication bias based on Begg’s funnel plot (*P* = 0.917) or Egger’s test (*P* = 0.669).

**Conclusions:**

Cruciferous vegetable intake might be inversely associated with pancreatic cancer risk. Because of the limited number of studies included in this meta-analysis, further well-designed prospective studies are warranted to confirm the inverse association between cruciferous vegetable intake and risk of pancreatic cancer.

## Background

Pancreatic cancer ranks as the fourth leading cause of death in the USA, with an expected 17,870 deaths in 2013 [1]. It carries a dismal prognosis with a 5-year survival rate of less than 5%, and for most patients, death occurs within 6 months after diagnosis of cancer [2]. The study of pancreatic cancer has assumed a position of growing importance because of the poor prognosis and increasing incidence in recent years [3]. The primary causes in pancreatic cancer have been poorly understood. Epidemiologic studies indicate a positive association between pancreatic cancer and cigarette smoking, diabetes mellitus, and body fatness [4,5]. At present, the role of diet in the prevention of pancreatic cancer remains unclear.

Fruits and vegetables contain numerous substances with potential anticarcinogenic activity and could therefore play a role in the prevention of pancreatic cancer [6]. Among specific subgroups of vegetables, cruciferous vegetables have been widely regarded as potentially cancer protective in recent years. Cruciferous vegetables are a group of vegetables named for their cross-shaped flowers, including broccoli, cauliflower, cabbage, and brussel sprouts and other members of the family. Previous meta-analyses have revealed that cruciferous vegetable intake is inversely associated with risk of breast cancer, kidney cancer, bladder cancer, and prostate cancer [7-10]. In epidemiological studies, the possible relationship between cruciferous vegetable intake and pancreatic cancer risk has also been investigated [11-14], but the findings are inconsistent, possibly as a result of the limited evidence and lack of statistical power in the individual studies.

The purpose of the present study was to summarize the evidence on the association between cruciferous vegetable intake and the risk of pancreatic cancer by conducting a meta-analysis on all relevant published epidemiological studies. To the best of our knowledge, this is the first meta-analysis regarding the relationship between cruciferous vegetable intake and pancreatic cancer risk.

## Methods

### Publication search

We conducted a literature search of published papers in March 2014 using PubMed, EMBASE, and Web of Science. We used the following search algorithm: (vegetables OR cruciferous OR brassica OR broccoli OR cauliflower OR cabbage) AND (pancreas OR pancreatic) AND (neoplasm OR cancer). All potentially relevant publications were evaluated by examining their titles and abstracts, and all of the studies matching the eligible criteria were retrieved. We also performed hand searches via cited references from retrieved articles and previous reviews on cruciferous vegetables and cancer. To be included in this meta-analysis, the studies had to meet all of the following criteria: (a) The exposure of interest was the intake of cruciferous vegetables, (b) the outcome of interest was pancreatic cancer, (c) the study had a case–control or cohort study design, and (d) the risk estimates with their corresponding 95% confidence intervals (CIs) were reported (or data were available to calculate these values). For publications with same population resources or overlapping datasets, the study with the largest number of cases was included in the meta-analysis. This systematic review was planned, conducted, and reported in adherence to the standards of quality for reporting meta-analyses [15].

### Quality assessment

We assessed the quality of all included studies by the Newcastle-Ottawa Scale (NOS) (http://www.ohri.ca/programs/clinical_epidemiology/oxford.asp). NOS is an eight-item instrument that allows for the assessment of the patient population and selection, study comparability, follow-up, and the outcome of interest. Interpretation of the scale is performed by awarding points, or ‘stars’, for high-quality elements. The stars are then added up and used to compare study quality in a quantitative manner. The maximum score could be 9 points, representing the highest methodological quality. We assigned scores of <7 and ≥7 for low- and high-quality studies, respectively. Any disagreements were resolved by a joint re-evaluation of the original article with a third reviewer.

### Data extraction

We extracted the following data from each study: the first author’s name, year of publication, the country in which the study was carried out, study design, study period, age of patient, sample size, exposure range, potential confounding variables adjusted, and exposure assessment. The relative risk (RR) was assumed to be approximately equivalent to the odds ratio (OR), and the OR was used as the study outcome. Data were extracted independently by two investigators using a predefined data collection form, and discrepancies were resolved by consensus, with involvement of a third reviewer when necessary.

### Statistical methods

Study-specific ORs and their 95% CIs for highest versus lowest intake of cruciferous vegetable level were extracted from each article. We pooled data using the fixed or random effects models depending on heterogeneity between studies in our meta-analysis. Between-study heterogeneity across the eligible comparisons was quantitatively assessed using the *Q* statistical test and *I*^2^ score [16]. Heterogeneity was confirmed with a significance level of *P* ≤ 0.10. The Galbraith plot was also used to detect the possible sources of heterogeneity [17], and a re-analysis was conducted with exclusion of the studies possibly causing the heterogeneity. The subgroup analyses were carried out by study design, study region, study quality, and whether controlling for smoking, body mass index (BMI), or diabetes mellitus (DM). We conducted sensitivity analyses by excluding each study at a time from the meta-analysis. Cumulative meta-analysis was also conducted by sorting the studies based on publication time. We assessed publication bias using the tests of Egger [18] and Begg [19]. We also used the trim-and-fill method to evaluate publication bias [20]. Statistical significance was considered while *P* < 0.05. All statistical analyses were done with Stata statistical software, version 11.0.

## Results

### Literature search and study characteristics

**Figure 1:**
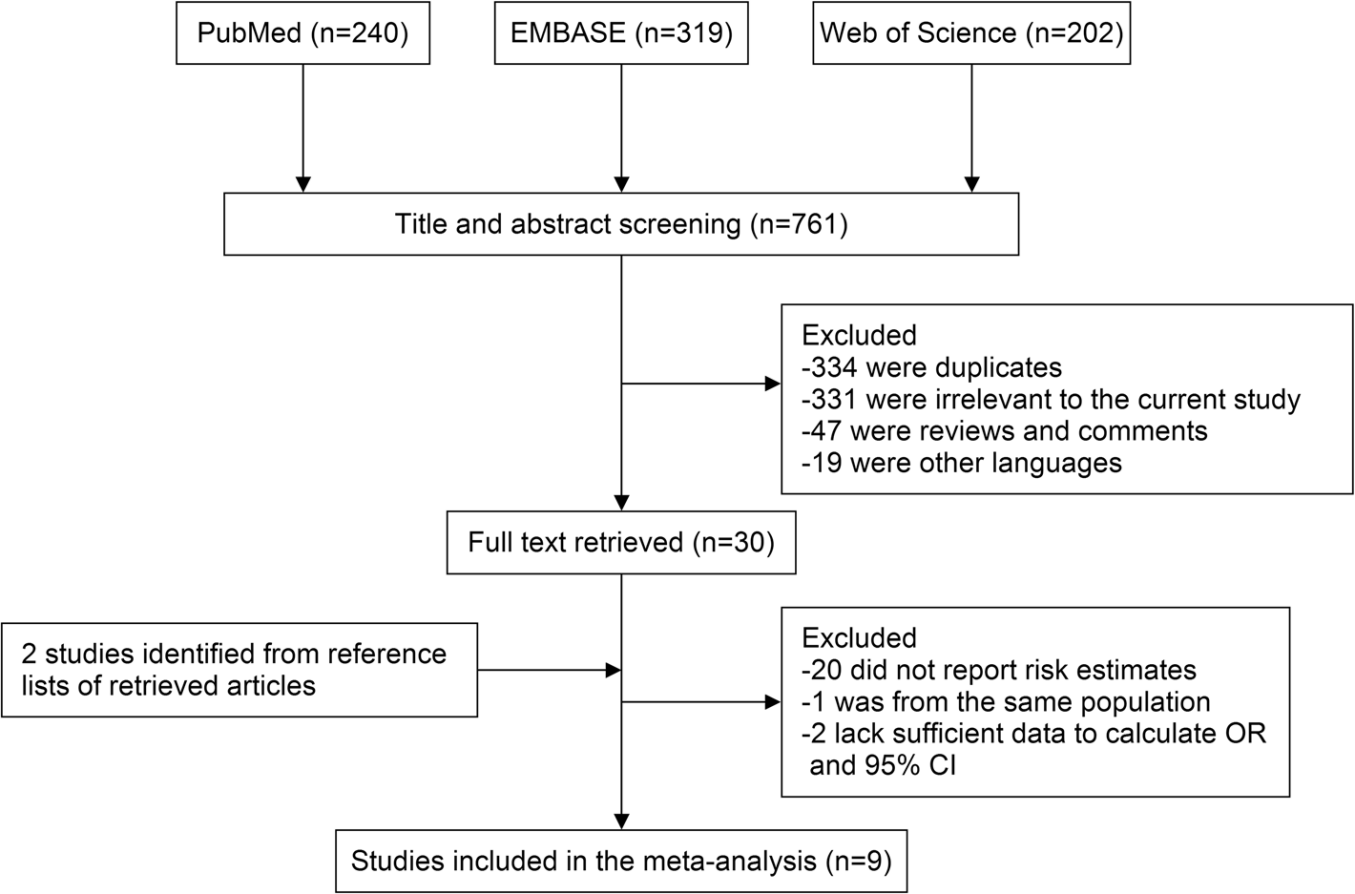
**Process of study selection.**

The process of identifying and selecting studies is shown in Figure 1. A total of nine studies were eventually recruited in this meta-analysis on the association of cruciferous vegetable intake with pancreatic cancer risk [7,8,12,21-26]. These studies (four cohort and five case–control studies) were conducted in the following regions: North America (*n* = 4), Europe (*n* = 4), and China (*n* = 1). All of the included studies were published between 1989 and 2012 and involved a total of 3,207 cases. Information on cruciferous vegetable intake was obtained by interview or a self-administered questionnaire. The study quality scores, assessed by the NOS, ranged from 4 to 8 (with a mean of 6.3). Table 1 presents the basic characteristics of each study included in our meta-analysis.

**Table 1:** **Study characteristics of published cohort and case–control studies on cruciferous vegetable intake and pancreatic cancer**

**Authors**	**Publication year**	**Study design**	**Study period**	**Country**	**Age**	**Cases/subjects**	**Exposure range**	**Study quality**	**Variables of adjustment**	**Cruciferous vegetable assessment**
Olsen et al.	1989	Population-based case–control study	1980–1983	USA	40–84	212/432	≥9 vs ≤2 times/month	5	Age, education level, reported diabetes mellitus history, cigarette smoking, alcohol, and when appropriate meat and/or vegetable consumption	Interview
Ji et al.	1995	Population-based case–control study	1990–1993	China	30–74	451/2,003	Highest vs lowest quartile	4	Age, income, smoking, green tea drinking (females only), and response status	Interview
Silverman et al.	1998	Population-based case–control study	1986–1989	USA	30–79	436/2,439	Highest vs lowest quartile	8	Age at diagnosis/interview, race, study area, calories from food, diabetes, cholecystectomy, body mass index, cigarette smoking, alcohol consumption, income, and marital status	Interview
Stolzenberg-Solomon et al.	2002	Cohort	1985–1997	Finland	50–69	163/27,111	>22.7 vs ≤1.8 g/day	7	Age, years of smoking, and energy intake	Questionnaire
Chan et al.	2005	Population-based case–control study	1995–1999	USA	21–85	532/2,233	Highest vs lowest quartile	6	Age, sex, and energy intake	Interview
Larsson et al.	2006	Cohort	1987–1997	USA	45–79	135/81,922	≥3 vs <1 servings/week	7	Age, sex, education, body mass index, physical activity, cigarette smoking status and pack-years of smoking, history of diabetes, multivitamin supplement use, and intakes of total energy and alcohol	Questionnaire
Nothlings et al.	2006	Cohort	1993–2002	German	45–75	529/183,522	Highest vs lowest quintile	7	Age, sex, age at cohort entry, ethnicity, history of diabetes mellitus, family history of pancreatic cancer, smoking status, pack-years of smoking, intakes of red meat and processed meat, energy intake, and body mass index	Questionnaire
Heinen et al.	2012	Cohort	1996–1997	Netherlands	55–69	423/120,852	>41.8 vs ≤10.6 g/day	7	Age, sex, smoking, body mass index, family history of pancreatic cancer, history of diabetes mellitus, intake of energy, red meat, coffee, and alcohol	Questionnaire
Bosetti et al.	2012	Hospital-based case–control study	1991–2009	Italy	63 (median)	326/978	≥1 vs <1 portions/week	6	Age, sex, study center, year of interview, education, body mass index, alcohol drinking, tobacco smoking, and total energy intake	Interview

### High versus low cruciferous vegetable intake

**Figure 2:**
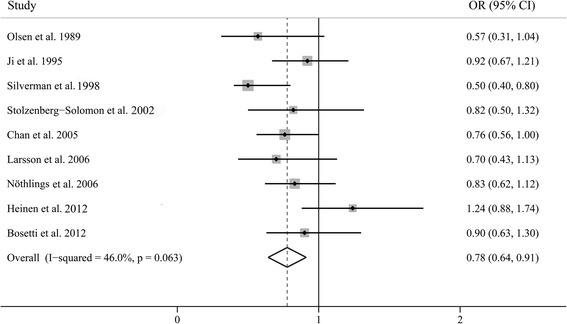
**A forest plot showing risk estimates from case–control and cohort studies estimating the association between cruciferous vegetable intake and pancreatic cancer risk.**

The multivariable-adjusted ORs of the highest versus lowest categories of cruciferous vegetable intake, for each study and for the combination of all of the studies, are shown in Figure 2. We found a significantly decreased risk of pancreatic cancer associated with a high intake of cruciferous vegetables (OR 0.78, 95% CI 0.64–0.91).

Next, we performed subgroup analyses by study design, study quality, and geographical region (Table 2). In the subgroup analyses separated by study design, the observed associations were more pronounced in the case–control studies (OR 0.72, 95% CI 0.55–0.89) than in the cohort studies (OR 0.87, 95% CI 0.67–1.06). When we stratified by study quality, the ORs (95% CI) were 0.78 (0.55–1.01) for high-quality studies and 0.80 (0.66–0.94) for low-quality studies. Furthermore, when separately analyzed by geographical area, the summary ORs (95% CI) for the USA, Europe, and China were 0.62 (0.49–0.76), 0.91 (0.74–1.07), and 0.92 (0.67–1.21), respectively. We also investigated the impact of some confounding factors on the estimates of ORs (Table 2). Smoking, diabetes mellitus, and BMI are potential confounders of the relationship between cruciferous vegetable intake and the risk of pancreatic cancer. Among the eight studies that controlled for smoking, the pooled OR was 0.79 (95% CI 0.63–0.94). When further separated and analyzed by DM, the ORs (95% CI) were 0.76 (0.56–0.96) for studies that controlled for DM and 0.82 (0.67–0.98) for studies that did not control for DM. Moreover, the summary effect estimates for studies that adjusted for BMI or not were 0.80 (95% CI 0.57–1.03) and 0.78 (0.64–0.93), respectively.

**Table 2:** **Summary of pooled risk estimates of pancreatic cancer in subgroups**

**Subgroup**	**No. of studies**	**Summary OR (95% CI)**	***Q*** **-test for heterogeneity**	
			***I*** ^**2**^ **score (%)**	***P*** **value**
All studies	9	0.78 (0.64, 0.91)	46.0	0.063
Study design				
Cohort	4	0.87 (0.67, 1.06)	23.0	0.273
Case–control	5	0.72 (0.55, 0.89)	53.1	0.074
Study quality				
High	5	0.78 (0.55, 1.01)	64.4	0.024
Low	4	0.80 (0.66, 0.94)	0	0.431
Geographical region				
USA	4	0.62 (0.49, 0.76)	6.5	0.36
Europe	4	0.91 (0.74, 1.07)	0	0.416
China	1	0.92 (0.67, 1.21)	-	-
Control smoking				
Yes	8	0.79 (0.63, 0.94)	52.7	0.039
No	1	0.76 (0.56, 1.00)	-	-
Control DM				
Yes	6	0.76 (0.56, 0.96)	60.8	0.026
No	3	0.82 (0.67, 0.98)	0	0.667
Control BMI				
Yes	5	0.80 (0.57, 1.03)	67.0	0.017
No	4	0.78 (0.64, 0.93)	0	0.499

### Evaluation of heterogeneity

**Figure 3:**
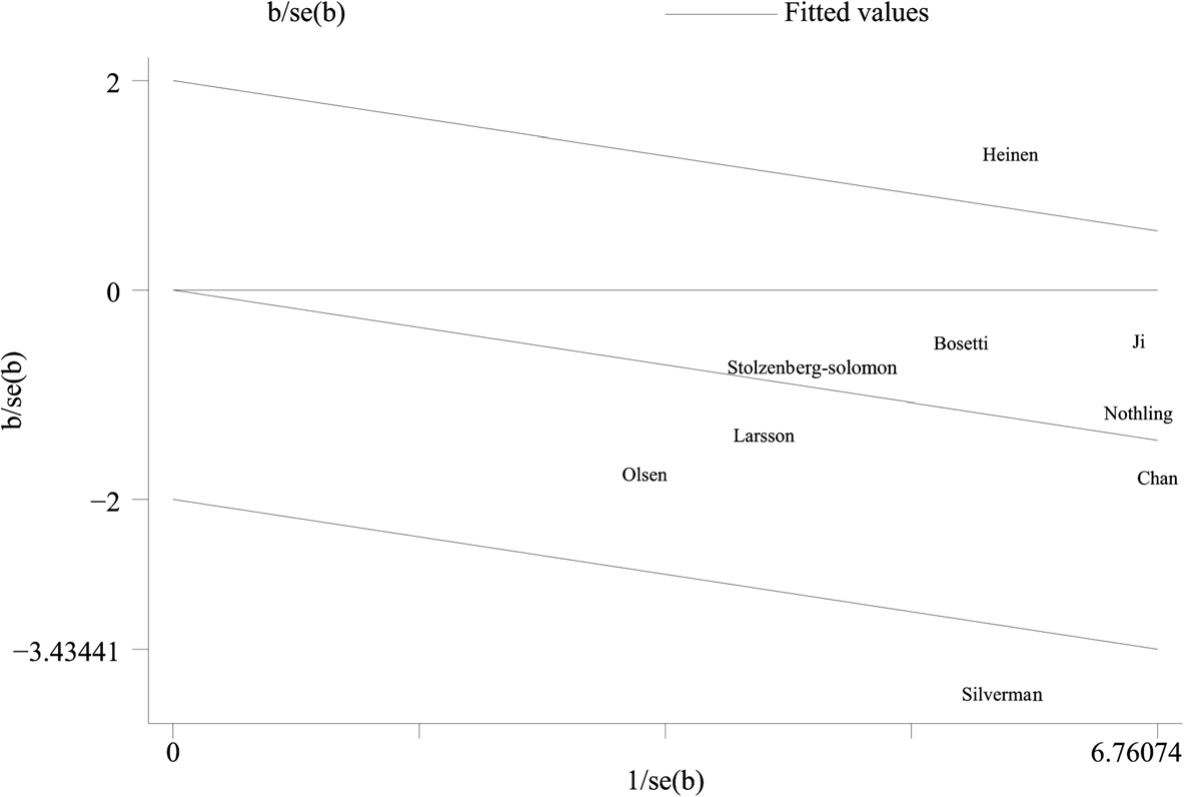
**Galbraith plot analysis indicated that two studies were the potential source of heterogeneity.**

In this meta-analysis, we used the *Q* test and the *I*^2^ index to evaluate the heterogeneity across studies. As shown in Figure 2, there was moderate heterogeneity among the studies (*P* = 0.065 for heterogeneity, *I*^2^ = 46.0%). Through the Galbraith plot (Figure 3), we found that two studies by Heinen et al. [12] and Silverman et al. [23] were the major sources of heterogeneity. After excluding these two studies, there was no study heterogeneity (*P* = 0.793, *I*^2^ = 0.0%), but the overall association was not significantly changed (OR 0.80, 95% CI 0.69–0.91).

### Sensitivity analysis

In the sensitivity analysis, the influence of each study on the pooled OR was examined by repeating the meta-analysis while omitting each study, one at a time. The study-specific ORs ranged from a low of 0.73 (95% CI 0.63–0.82) to a high of 0.82 (95% CI 0.72–0.93) via the omission of the study by Heinen et al. [12] and the study by Silverman et al. [23], respectively.

### Cumulative meta-analysis

**Figure 4:**
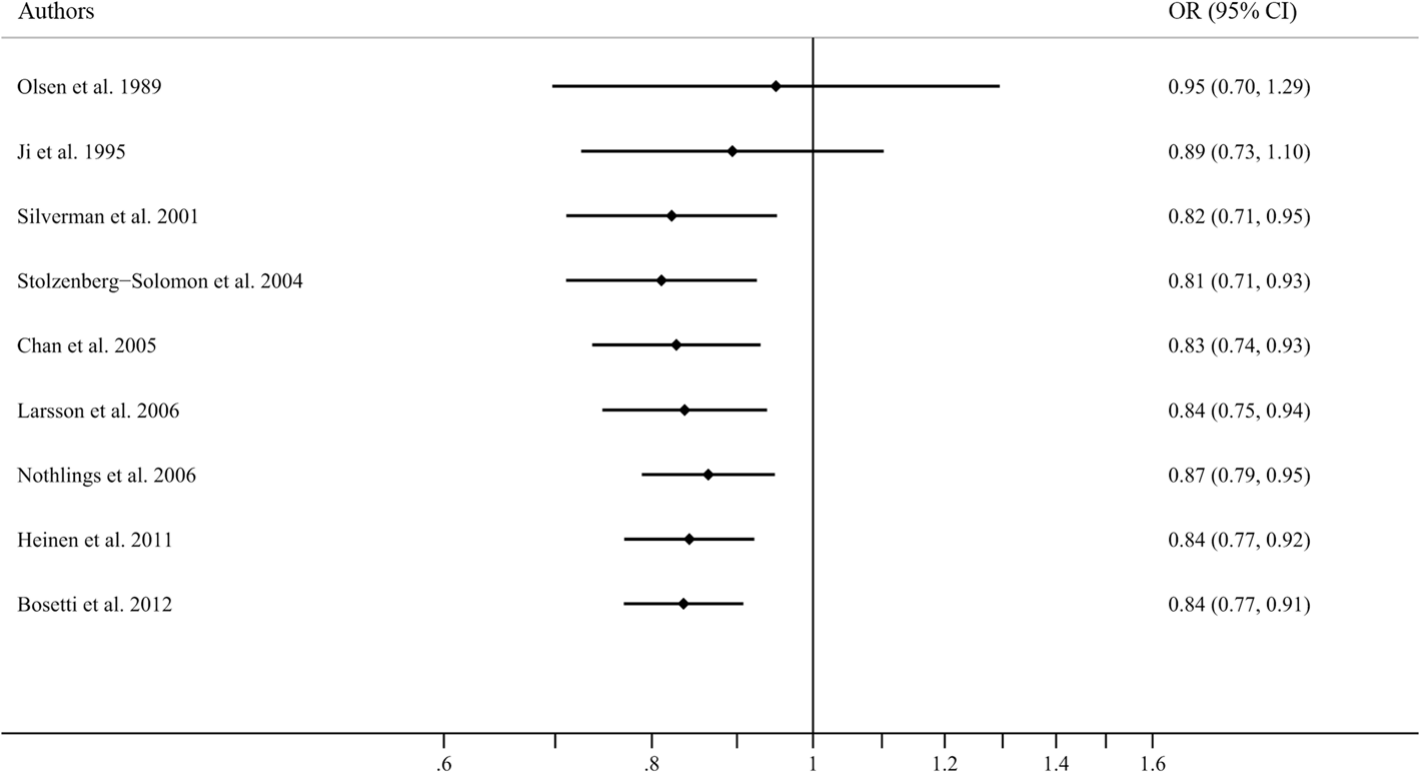
**A forest plot showing cumulative meta-analysis of cruciferous vegetable intake and pancreatic cancer risk.**

Cumulative meta-analysis was also conducted by sorting the studies based on publication time. Figure 4 shows the results from the cumulative meta-analysis of the association between cruciferous vegetable intake and pancreatic cancer risk in chronologic order. The 95% CIs became increasingly narrower with the increasing sample size, indicating that the precision of the estimates was progressively boosted by the continual addition of more cases.

### Publication bias

**Figure 5:**
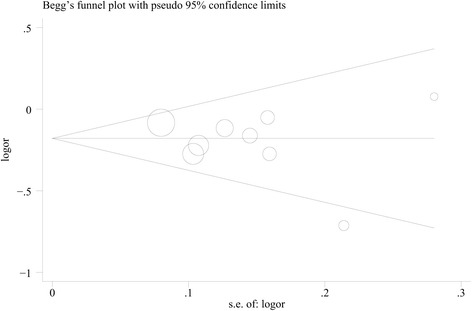
**Funnel plot of cruciferous vegetable intake and pancreatic cancer risk.**

There was no evidence of significant publication bias with Begg’s funnel plot (Figure 5, *P* = 0.917) or with Egger’s test (*P* = 0.669). The trim-and-fill analysis identified one possible missing study that would not have altered our results (OR 0.82, 95% CI 0.74–0.91).

## Discussion

Cruciferous vegetables have been studied extensively aiming to evaluate their chemopreventive properties. Although several meta-analyses have suggested that cruciferous vegetables have been associated with reduced risks of several other cancers [7-10], little is known about the effects of cruciferous vegetable intake on pancreatic cancer risk. A few previous studies have been conducted to assess the relationship between them, but the results were inconsistent. As individual studies may have insufficient statistical power, our meta-analysis of nine studies involving a relatively large number of cases and participants enhanced the power to detect a significant association and provided more reliable estimates. To the best of our knowledge, this is the first meta-analysis evaluating the relationship between cruciferous vegetable intake and incidence of pancreatic cancer. The results indicated that high cruciferous vegetable consumption might be associated with low risk of pancreatic cancer (OR 0.78, 95% CI 0.64–0.91).

The observed heterogeneity among the studies of cruciferous vegetable intake and pancreatic cancer risk seemed to be explained by the studies of Heinen et al. [12] and Silverman et al. [23], which reported the positive and strongest protective relationships, respectively. After excluding these two studies, the association between cruciferous vegetable intake and the risk of pancreatic cancer was not significantly changed. In the subgroup analysis, the pooled analysis from the case–control studies suggested an obvious reduction in risk; the results from the cohort studies were non-significant, suggesting that our conclusion depends mainly on the case–control studies. However, the summary RR for cohort studies was close to being significant, and it can be speculated that cruciferous vegetable intake is likely to be associated with decreased risk of pancreatic cancer if more cohort studies were included. In the subgroup analysis separated by geographical regions, the association was stronger for studies from the USA (OR 0.62, 95% CI 0.49–0.76) than Europe (OR 0.91, 95% CI 0.74–1.07) and China (OR 0.92, 95% CI 0.67–1.21), suggesting that regional difference may contribute to the observed heterogeneity. We also examined some important confounding factors, including smoking, BMI, and diabetes. It has been established that cigarette smoking is one of the most important risk factors for developing pancreatic cancer [27]. Recently, a number of epidemiological studies and system reviews have reported that BMI and DM are associated with an increased risk of pancreatic cancer [28-31]. High cruciferous vegetable intake tends to be associated with healthy behaviors, which are related to lower body mass index and reduced risk of diabetes. Nevertheless, the inverse relationship still persisted after combining the studies adjusted for smoking, BMI, and diabetes, further confirming the reliability of the results of our study that cruciferous vegetable intake is likely a protective factor against pancreatic cancer.

A preventive role of cruciferous vegetables in the development of pancreatic cancer is plausible. Isothiocyanates, a constituent of cruciferous vegetables, have been hypothesized to induce xenobiotic-metabolizing enzymes, which are involved in eliminating potential DNA carcinogens [32]. Sulforaphane, which is found in broccoli and broccoli sprouts at particularly high levels, has been the most extensively studied. The potent antiproliferative activity of sulforaphane was observed in a panel of cultured human pancreatic cancer cells [33,34]. Kallifatidis et al. [35] showed that sulforaphane in combination with TRAIL may be a promising strategy for targeting treatment-resistant pancreatic tumor-initiating cells. Additionally, benzyl isothiocyanate and phenethyl isothiocyanate have also been shown to inhibit proliferation and induce apoptosis in pancreatic cancer cells *in vitro* and *in vivo* [36,37].

There are several important limitations to be considered in interpreting the results of our meta-analysis. First, our meta-analysis only included published studies in English; limited resources prevented us from including articles published in other languages. In addition, our search was restricted to studies published in indexed journals. We did not search for unpublished studies or original data. The small number of published studies severely limited the ability to detect publication bias, although our results seem to suggest that there was no evidence of publication bias. The results of funnel plot and Egger’s test still should be interpreted cautiously. Furthermore, it is known that in Asia, people consume large amounts of cruciferous vegetables, and this is an ideal population to study their action in health. However, only one study from China was included in this meta-analysis. Second, we assessed total cruciferous vegetable consumption because of the relatively large number of studies on the topic. However, cruciferous vegetables include a group of vegetables such as broccoli, cauliflower, cabbage, and brussel sprouts and other members of the family. Our pooled estimates represented the combination of different types of cruciferous vegetables that may bring about different effects on pancreatic cancer. Third, the lower risk estimates when consumption is evaluated in several categories could be explained if cruciferous vegetables had a protective effect that would be stronger when the difference between the groups of the highest and lowest exposure was larger. Therefore, only two exposure levels (the highest and lowest cruciferous vegetables) were examined in our meta-analysis. There was a wide range of values for the cutoff points for the lowest and highest categories of cruciferous vegetable intake in the included studies, which have contributed to the heterogeneity among studies in the analysis.

## Conclusions

Cruciferous vegetable intake might be inversely associated with the incidence of pancreatic cancer. Because of the limited number of studies, further experimental and well-designed prospective epidemiologic studies are needed to affirm the findings from our study.

## Abbreviations

CI: confidence interval
OR: odds ratio
RR: relative risk
